# Supplementary material for: The interactome of intact mitochondria by cross-linking mass spectrometry provides evidence for coexisting respiratory supercomplexes
Source: Mol Cell Proteomics. 2017 Dec 8;17(2):216–32. doi: 10.1074/mcp.RA117.000470 (PMC5795388; doi:10.1074/mcp.RA117.000470)
Supplement: Supplemental Data [file supp_RA117.000470_133922_0_supp_23978_fzffwf.zip › spectra_annotation/mito_spectra_annotation/72-1-10-1-160-1.pdf]

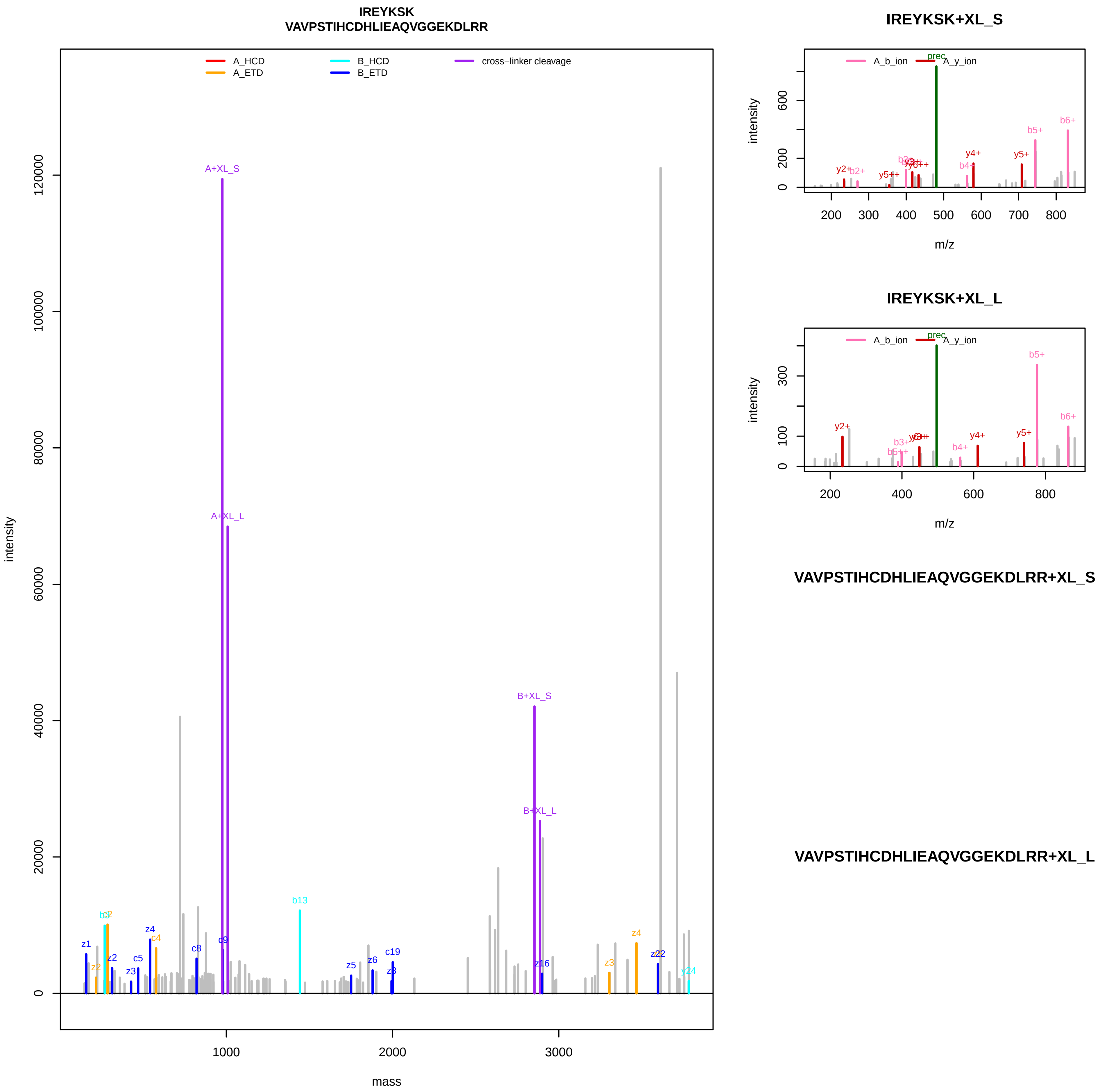

IREYKSK
VAVPSTIHCDHLIEAQVGGEKDLRR
A_HCD
A_ETD
B_HCD
B_ETD
cross−linker cleavage
A+XL_S
A+XL_L
B+XL_S
B+XL_L
b3
z2
z1
z2
z3
c5
z4
c4
c8
c9
b13
z5
z6
c19
z3
z16
z3
z4
z22
y24
intensity
mass
0
20000
40000
60000
80000
100000
120000
1000
2000
3000
IREYKSK+XL_S
A_b_ion
A_y_ion
prec
y2+
b2+
y5++
b3+
b4+
y4+
y5+
b5+
b6+
m/z
IREYKSK+XL_L
A_b_ion
A_y_ion
prec
y2+
b3+
b5++
b4+
y4+
y5+
b5+
b6+
m/z
VAVPSTIHCDHLIEAQVGGEKDLRR+XL_S
VAVPSTIHCDHLIEAQVGGEKDLRR+XL_L
